# Successful Administration of Kampo Medicine and Acupuncture Treatment to Improve Erythromelalgia: A Case Report

**DOI:** 10.7759/cureus.65890

**Published:** 2024-07-31

**Authors:** Keisuke Kumada, Jun Matsumoto-Miyazaki, Hideshi Okada, Hiroyuki Okura, Yasumasa Sato

**Affiliations:** 1 Patient Safety Division, Gifu University Hospital, Gifu, JPN; 2 Department of Cardiology and Respirology, Gifu University School of Medicine, Gifu, JPN; 3 Department of Emergency & Disaster Medicine, Gifu University School of Medicine, Gifu, JPN; 4 Department of Obstetrics and Gynecology, Gifu Prefectural General Medical Center, Gifu, JPN

**Keywords:** visual analog scale (vas), traditional chinese medicine, erythromelalgia, kampo medicine (japanese herbal medicine), acupuncture treatment

## Abstract

Erythromelalgia is a rare disease characterized by a triad of recurrent burning pain, redness with pain, and hot flashes in the legs during attacks. We report the case of a 40-year-old woman who suffered from refractory erythromelalgia for 15 years and was successfully managed with Kampo medicine and acupuncture.

Her pain was refractory to seven types of oral medications and intravenous lidocaine injections. Byakkokaninjinto was also administered for concomitant polydipsia in addition to acupuncture, unseiin, orengedokuto, and sokeikakketsuto. Because erythromelalgia has no established treatment, traditional Kampo medicine combined with acupuncture may help improve the quality of life of affected patients.

## Introduction

Erythromelalgia affects patients intermittently and bilaterally, more often in the lower limbs. It is characterized by a triad of recurrent burning pain, redness with pain, and hot flashes in the legs during attacks, which worsen with exercise and high temperatures [1]. The pain can be so severe that patients may engage in behaviors that are sometimes extreme to cool the affected areas and change their lifestyle to avoid precipitating factors such as exercise and increased ambient heat. However, the pain can be alleviated by cooling the affected area. Because erythromelalgia has no established treatment, treatment usually consists of symptomatic pain management. Herein, we report a case of a woman whose symptoms of refractory erythromelalgia were improved with traditional Japanese medicine (Kampo medicine) and acupuncture.

## Case presentation

History of present illness

A 40-year-old woman with a 15-year history of erythromelalgia presented with a tingling sensation distal to the lower extremities. She had been previously managed with opioids at the anesthesiology and pain department for pain and hot flashes in the extremities. Despite receiving regular pain relievers, her pain was not ameliorated, and she was subsequently referred to our department for the chief complaints of hotness and pain in the limbs and polydipsia. We began oral administration of duloxetine hydrochloride 40 mg/day, trazodone hydrochloride 25 mg/day, alprazolam 0.4 mg/day, codeine phosphate hemihydrate 800 mg/day, triazolam 0.25 mg/day, pregabalin 300 mg/day, morphine hydrochloride 20 mg/day, dydrogesterone 10 mg/day, Jiinshihoto (JST) 7.5 g/day, and naldemedine tosylate 2 mg/day, and 2% lidocaine injection was administered intravenously once every 2 weeks.

Medical history

There was a previous history of psychiatric examination for suicidal ideation.

Clinical characteristics

**Figure 1:**
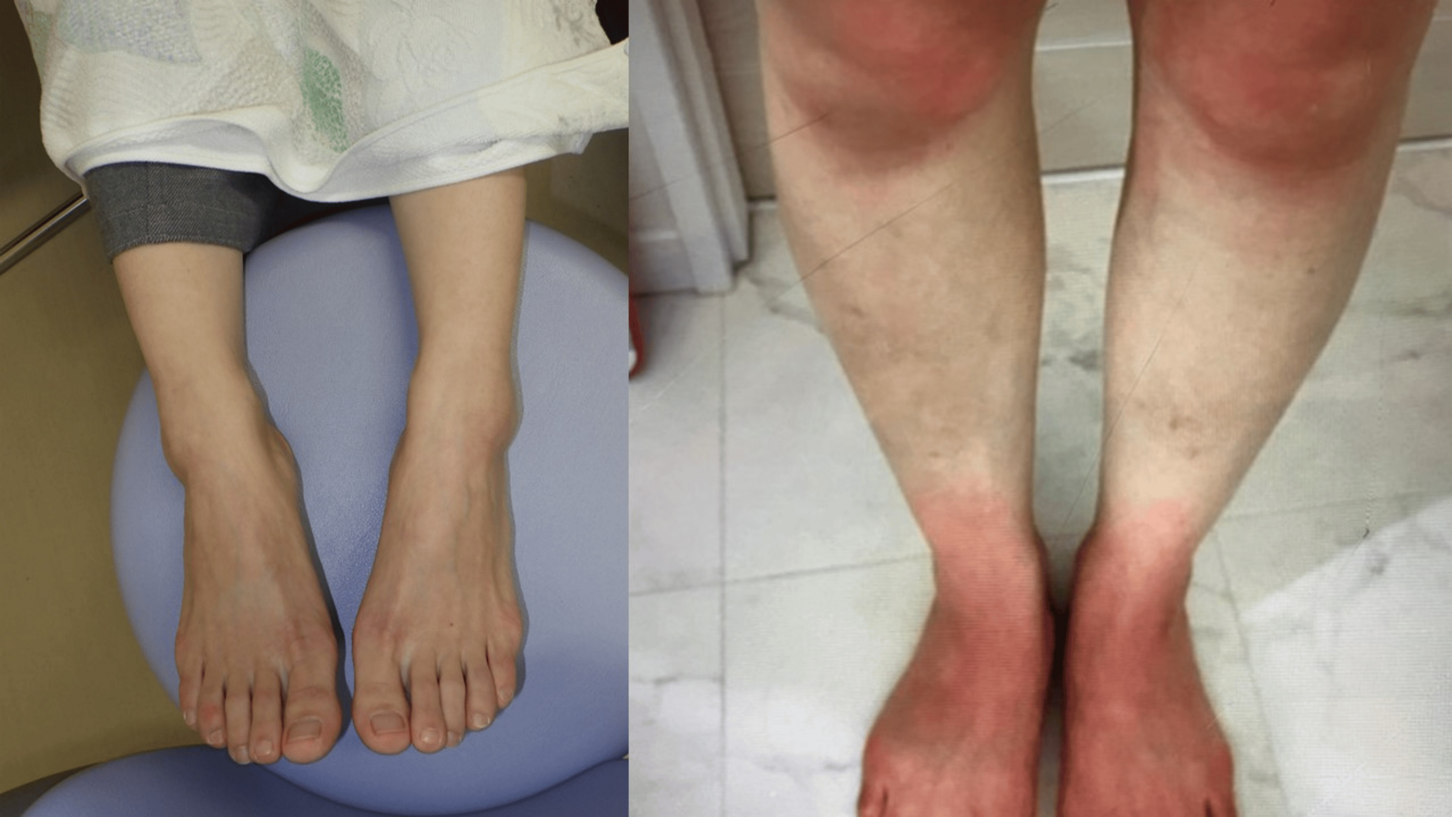
Changes in the color of the legs in a normal state and during symptom onset The patient normally has no symptoms (left), but redness with pain and hot flashes in the legs are observed during attacks (right).

The patient reported intermittent redness accompanied by pain and hot flashes in her extremities, hands, and sometimes, the nipples and nose from night to early morning (Figure 1).

These symptoms also occurred after daily activities such as during housework, thermal stimulation caused by exertion, and physical stimulation. Some improvements in symptoms were observed with cooling and elevating the legs. The patient walked using a cane to reduce the load on her feet, was unable to take baths because of pain exacerbation by warm stimulation, and reported an extremely dry mouth, drinking several liters of water per day. The patient reported insomnia, nightmares, and night sweats in addition to irritability. When she visited our department, she complained of nausea and vomiting, overeating, and hard stools once every four days. At that time, it had been three years since her last menstrual cycle. During menstruation, her symptoms included pain, erythema, and hyperthermia, exacerbating before menstruation. There was no history of pregnancy. Her vital signs were as follows: blood pressure, 95/71 mmHg; heart rate, 78 beats per minute; and axillary temperature, 36.9°C

Clinical course

Based on traditional Kampo medicine and traditional Chinese medicine (TCM) theory, we speculated that an excessive internal heat pattern and blood stasis had induced the burning pain, erythema, and hyperthermia of extremities. Moreover, yin deficiency and excessive heat pattern had also been speculated to induce nocturnal exacerbations of her symptoms such as warmth and erythema. Qi stagnation and transforming internal fire and heart fire have also been thought to be related to the exacerbation of excessive internal heat.

We started the Kampo treatment that included Byakkokaninjinto (BKTN; extract TJ-34, Tsumura & Co, Japan), Unseiin (USI; extract TJ-57, Tsumura & Co), Orengedokuto (OGT; extract TJ-15, Tsumura & Co), and Sokeikakketsuto (SOKT; extract TJ-53, Tsumura & Co) based on Kampo and TCM theory and a report on Western medical theories of erythromelalgia mechanism. Furthermore, two months after the commencement of BKTN, acupuncture treatment was initiated.

**Figure 2:**
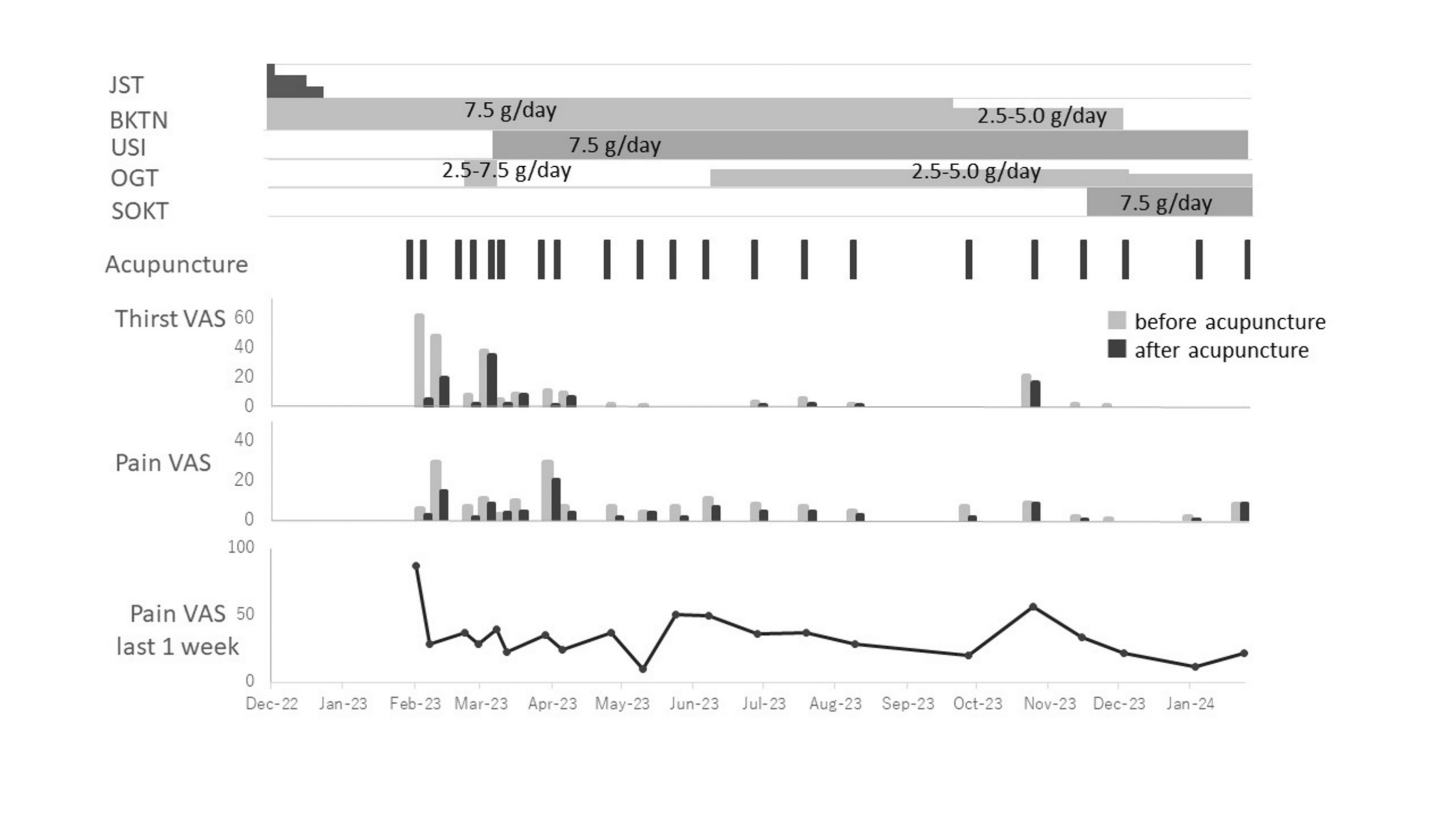
Clinical course Thirst and pain were evaluated with the VAS at the time of acupuncture treatment. JST, Jiinshihoto; BKTN, Byakkokaninjinto; USI, Unseiin; OGT, Orengedokuto; SOKT, Sokeikakketsuto; VAS, visual analog scale

Pain and thirst before and immediately after each acupuncture session were evaluated using a 100-mm visual analog scale (VAS) in which the left and right endpoints of the VAS indicated no symptom and the greatest symptom intensity imaginable, respectively. Pain intensity during the last week was also evaluated using VAS before each acupuncture session because her pain exacerbated at night even if it was not severe in the daytime during her visit to our hospital (Figure 2).

The patient was administered BKTN 9.0 g/day for the relief of severe heat patterns in TCM theory. JST prescribed by the previous hospital was weaned off considering the use of yin-tonifying medicine might be tough on the patient because of spleen Qi deficiency, suspected due to digestive symptoms such as nausea, vomiting, and constipation. From day 10 of the prescribed treatment and medications in our department, the patient reported a significant reduction in thirst and hot flashes on her hands. Her pain, which was especially exacerbated at night, remained, suggesting that yin deficiency and blood stasis were sustained. However, adding another Kampo medicine was difficult because of the nausea and vomiting induced by the spleen qi deficiency. Therefore, we started acupuncture treatment to modulate spleen qi, increase yin, clear heat, and resolve qi stagnation and blood stasis.

Acupuncture was started two months after BKTN initiation. At the start of the acupuncture, her TCM findings included redness of the tongue, darkened and distended sublingual veins, thin and string-like pulse, tenderness of SP6, BL15, BL17, LR3, sweating through KI6 and KI3 of the skin. The abdominal findings included tension and tenderness in the pericardial and CV12 as well as in the costal and lateral abdomen. Acupuncture points, including SP6, KI6, KI7, GV20, BL15, BL17, and BL23, were commonly used. PC7 was added from the fourth to the seventh sessions of acupuncture, and LR3 was added from the seventh session. ST44 was added in the fifth session. Unilateral sides of these acupuncture points with a high degree of tenderness were used. The locations of the acupoints were determined according to the WHO standard textbook. These points were mainly selected based on the TCM theory and acupoint findings. SP6 and BL17 were used for activating blood and resolving stasis. KI6, KI7, and BL23 were used for nourishing yin and clearing heat. GV20, LR3, and PC7 were used to relieve Qi stagnation and sedate the heart and spirit, and SP6 was also used for spleen Qi deficiency. Disposable stainless steel acupuncture needles (0.12 or 0.16 mm in diameter, 40 mm in length SEIRIN, Shizuoka, Japan) were gently inserted to a depth of 2-4 mm on each point and left for 7 min without manipulation. The needle at KI6 was inserted horizontally; however, at other acupoints, the needles were inserted perpendicularly.

The patient reported a considerable reduction in thirst during the first acupuncture session (VAS, from 62 to 5). Moreover, her pain exacerbation at night tended to decrease after the initiation of acupuncture. Her pain one week after the first acupuncture session was reduced compared with the pain one week before the first session (VAS, from 88 to 29). The patient reported less pain at night during the first three days after each acupuncture session. Although the pain before the second acupuncture session was relatively high, it reduced immediately after acupuncture therapy (VAS from 29 to 15). Nausea and vomiting were also relieved after the start of the acupuncture therapy. Her basal body temperature reduced from 37.1 °C to 36.5 °C. Night sweats and nightmares were also resolved after two acupuncture sessions. The acupuncture therapy was continued thereafter, at intervals of two or three weeks (in total, 21 sessions for 51 weeks).

Three months later, she reported insomnia from heat sensation in the extremities with increased body temperature in spring, irritability, and nose bleeding. OGT was added to further strengthen the clearing heat effect; however, it was changed to USI, which is a combination product of OGT and Shimotsuto (SMT), because of skin dryness.

Seven months later, the instances of the patient waking up due to pain in the middle of the night came under control (VAS, from 29 to 21), her menstrual cycles resumed, consuming regular meals became possible, and she had regular bowel movement once every 2 days without the use of laxatives.

After USI was continued and taken regularly, OGT was added whenever needed to reduce pain (2.5-5.0 g/day), and BKTN was tapered off.

Eleven months later, her symptoms recurred (VAS from 34 to 57); thus, SOKT (7.5 g/day) was prescribed to promote the relief of blood stasis, blood deficiency, and stasis of body fluid. After that stabilized the pain. Alprazolam, pregabalin, and dydrogesterone were discontinued, and her treatment included reducing morphine hydrochloride and codeine phosphate hemihydrate to 10 and 400 mg, respectively. The pain VAS 14 months after the start of the treatments in our departments was 22. Activities of daily living, such as showering and light housework, became easier. She has not had suicidal thoughts, and aside from occasional nightmares and night sweats, the quality of her sleep has improved.

No adverse and anticipated events due to the Kampo medicine and acupuncture occurred.

## Discussion

Erythromelalgia was first reported in 1878, and the term was coined by Drenth and Michiels [1]. Although it has no clear diagnostic criteria, the condition is clinically diagnosed by the triad of signs and symptoms of recurrent burning pain, warmth, and redness of the extremities, which are triggered by heat or exercise and alleviated by cooling [2]. It may spread to the entire body or may be localized to the ears, cheeks, thighs, and vulva, without symptoms in the limbs. The pathophysiology of erythromelalgia involves blood flow stagnation caused by the swelling of the arteriolar endothelium, platelet aggregation, and involvement of the autonomic nervous system; however, the details are unknown. As erythromelalgia has no established treatment [3], current treatment methods focus on controlling symptoms and improving the quality of life. Voltage-gated sodium channel blockers, calcium channel α2δ subunit ligands, antiplatelet agents, vasodilators, tricyclic antidepressants, and serotonin reuptake inhibitors are some of the previously reported pharmacological treatments for erythromelalgia. Given the inconsistent effects of these medications, patients are often treated with multiple drugs [4].

Very few studies have reported the use of Kampo medicine for erythromelalgia treatment, except for studies reporting the use of Hachimijiogan, Keishikaryukotsuboreito, and SOKT. Our patient had a chronic primary disease of >15 years duration. Her symptoms worsened in the early hours of the morning and were systemic, with symptoms not only affecting her lower extremities but also her hands, nipples, and nose. She was also experiencing psychiatric symptoms such as suicidal ideation. Her pain was refractory to seven oral medications and the intravenous administration of lidocaine.

Kampo medicine emphasizes patterns, and treatment modality is selected according to the most problematic symptom at a given time [5]. Therefore, we initially focused on managing the patient’s thirst, followed by treating her extremity symptoms. Given the extreme thirst and small urine output, we prioritized lowering her body temperature by administering BKTN for its expected heat-clearing and enriched yin effects. In addition to these effects based on the TCM theory, gypsum, the main component of BKTN, acts on aquaporins 2 and 3 to lower the body temperature and regulate body fluid balance [6,7].

After the patient’s thirst was under better control and her water intake was reduced to 2 L a day, we treated the hot flashes in the extremities, chest, palms, and soles using USI, a combination product of OGT and SMT. Indications for USI include irregular menstrual cycles, dysmenorrhea, and neurosis; however, it is also effective for inflammatory skin diseases, having successfully treated intractable atopic dermatitis in some cases [8]. USI was also administered because OGT is effective for febrile diseases and mental symptoms [9], and SMT has pharmacodynamic effects such as improving peripheral circulation and an antiplatelet effect [10]. The aggravation of symptoms from night to dawn was attributed to not only yin deficiency but also blood stasis, which explained the effectiveness of SOKT, given its clearing heating and reliving blood stasis effects [11].

Acupuncture also reduced the pain in her extremities and thirst. To the best of our knowledge, there are no English reports regarding acupuncture for erythromelalgia. Reportedly, acupuncture is used to treat chronic pain caused by various diseases. Acupuncture can modulate neurotransmitters, including opioids, norepinephrine, serotonin, orexin, and endocannabinoids in the central nervous system to induce analgesic effects [12]. Acupuncture has also been reported to reduce cyclooxygenase-2 and prostaglandin E2 levels at the peripheral level, mediating peripheral opioid release [12]. These acupuncture-related effects might have reduced the patient’s pain.

In the patient, adding other Kampo medicines was thought to be difficult due to nausea and vomiting. Unlike herbal medicines, acupuncture does not burden digestion. Thus, the nausea and vomiting experienced by the present patient were relieved after initiating the acupuncture treatment. Moreover, acute analgesic effects of acupuncture were observed when the patient’s pain was relatively high. The effect of acupuncture on the pain experienced at night lasted 3 days according to the patient. Given these findings, adding acupuncture was beneficial for relieving the symptoms of the patient.

## Conclusions

The administration of Kampo medicine and acupuncture based on Kampo and TCM theory resulted in the alleviation of the pain experienced by the patient in her extremities, resumption of menstruation, reduction of psychological symptoms, and improvement in the quality of life. Combining Kampo medicine with acupuncture may be effective treatment for erythromelalgia. Further studies are warranted to confirm the effects of the combination therapy for erythromelalgia.

## References

[1] Mørk C, Kvernebo K (2000). Erythromelalgia--a mysterious condition?. Arch Dermatol.

[2] Tang Z, Chen Z, Tang B, Jiang H (2015). Primary erythromelalgia: a review. Orphanet J Rare Dis.

[3] Ma JE, Lee JU, Sartori-Valinotti JC, Rooke TW, Sandroni P, Davis MD (2023). Erythromelalgia: a review of medical management options and our approach to management. Mayo Clin Proc.

[4] Tham SW, Giles M (2018). Current pain management strategies for patients with erythromelalgia: a critical review. J Pain Res.

[5] Yu F, Takahashi T, Moriya J (2006). Traditional Chinese medicine and Kampo: a review from the distant past for the future. J Int Med Res.

[6] Aburada T, Ikarashi N, Kagami M (2011). Byakkokaninjinto prevents body water loss by increasing the expression of kidney aquaporin-2 and skin aquaporin-3 in KKAy mice. Phytother Res.

[7] Ikarashi N, Ogiue N, Toyoda E (2012). Gypsum fibrosum and its major component CaSO4 increase cutaneous aquaporin-3 expression levels. J Ethnopharmacol.

[8] Kobayashi H, Takahashi K, Mizuno N, Kutsuna H, Ishii M (2004). An alternative approach to atopic dermatitis: part I-case-series presentation. Evid Based Complement Alternat Med.

[9] Choi Y, Kim Y, Kwon O, Chung SY, Cho SH (2021). Effect of herbal medicine (Huanglian-jie-du granule) for somatic symptoms and insomnia in patients with Hwa-byung: a randomized controlled trial. Integr Med Res.

[10] Takiyama M, Matsumoto T, Sanechika S, Watanabe J (2021). Pharmacokinetic study of traditional Japanese Kampo medicine shimotsuto used to treat gynecological diseases in rats. J Nat Med.

[11] Kanai S, Taniguchi N, Higashino H (2003). Study of sokei-kakketu-to (shu-jing-huo-xue-tang) in adjuvant arthritis rats. Am J Chin Med.

[12] Lin JG, Kotha P, Chen YH (2022). Understandings of acupuncture application and mechanisms. Am J Transl Res.

